# A novel conservative treatment to reduce cardiac herniation following intrapericardial pneumonectomy

**DOI:** 10.1111/1759-7714.13355

**Published:** 2020-02-08

**Authors:** Yunping Lan, Bin Hu, Xiaozun Yang, Bo Tian, Xiaojun Yang, Qiang Li, Ting Wang, Qinghua Zhou, Yunxia Zuo

**Affiliations:** ^1^ Department of Anesthesiology, West China Hospital Sichuan University Chengdu China; ^2^ Intensive Care Unit Sichuan Provincial People's Hospital and Sichuan Academy of Medical Science, School of Medicine, University of Electronic Science and Technology of China Chengdu China; ^3^ Department of Thoracic Surgery Sichuan Cancer Hospital & Institute, Sichuan Cancer Center, the affiliated Cancer Hospital, School of Medicine, University of Electronic Science and Technology of China Chengdu China; ^4^ Sichuan Lung Cancer Institute, West China Hospital Sichuan University Chengdu China

**Keywords:** Cardiac herniation, pericardium, pneumonectomy, saline, shock

We have previously reported reduction of cardiac herniation following intrapericardial pneumonectomy with pleural perfusion of saline in 2018.^1^ Compared with reoperation to reintroduce the heart into the pericardium as an urgent procedure, including returning it to the lateral position with no‐surgical side down and injecting air into the surgical hemithorax,^2^, ^3^, ^4^ pleural perfusion can be a simple and effective conservative management to reduce cardiac herniation to cure this acute and severe postoperative complication in ICU. Most cases survived in the reports highlighted here. 2, 3, 4 It should be noted that this is a rare complication and we replicated the treatment of pleural perfusion in Ba‐Ma mini pigs.^5^ After a right pneumonectomy was performed, we removed an elliptical patch of pericardium from the radix pulmonis to the pericardial fat pad (Fig 1a). Changing the left lateral position to the supine position, cardiac herniation occurred with displacement of the heart through the pericardial defect and rotated to the right hemithorax (Fig 1b). A significant decline in arterial blood pressure accompanied with arrhythmia was observed (Fig 1c), and the animal went into a state of shock. It was returned to the left lateral position with no‐surgical side down and 1000 mL warm saline was injected into the surgical hemithorax to reduce the cardiac herniation (Fig 1d). Immediately, the blood pressure raised, the heart beat converted to sinus rhythm, and circulation improved. A computerized tomography (CT) scan was performed with the animal in a supine position, and thoracic CT (Fig 1e) revealed that abundant pleural fluid had occupied the surgical hemithorax, and its buoyancy, volume and pressure had forced the heart to rotate back into the pericardium. Again, cardiac herniation occurred after drainage of the pleural fluid (Fig 1f). In conclusion, this animal model revealed the surgical procedure and confirmed that conservative treatment was effective.

**Figure 1:**
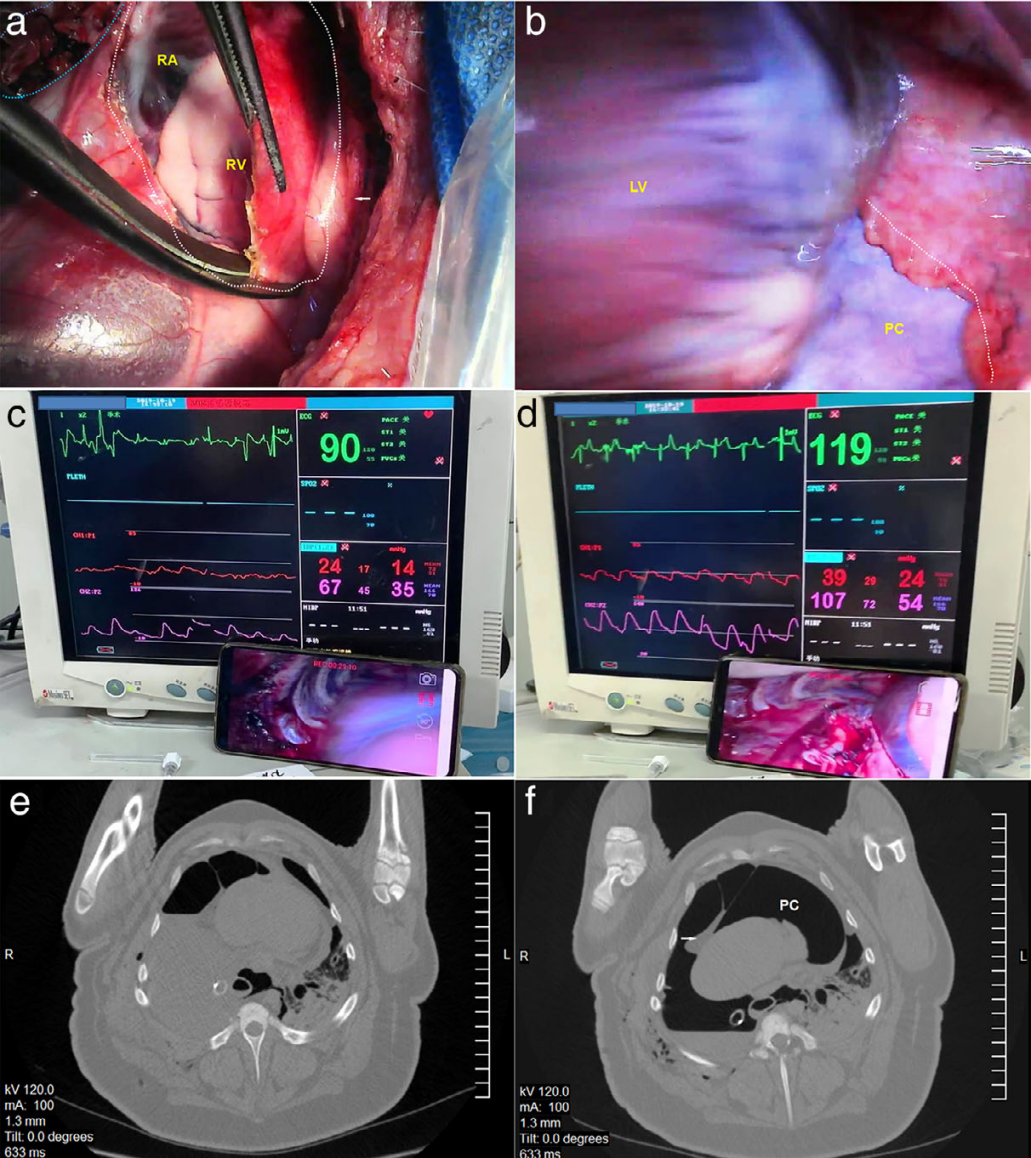
(**a**) A thoracoscopic view which shows the pericardial defect from the hilum to the pericardial fat pad, in left lateral position. (**b**) The heart was displaced through the pericardial defect and rotated to the right hemithorax, in supine position. (**c**) The monitor showed that the arterial blood pressure declined with arrhythmia when the animal was turned to a supine position. (**d**) The arterial blood pressure was raised and the heart converted to sinus rhythm after the animal was returned to a lateral position. (**e**) CT scan showed pleural fluid reduced the cardiac herniation, and the heart has rotated back into the pericardium. (**f**) After drainage of the pleural fluid, CT scan showed that the heart had spun anticlockwise and was located in the right chest cavity; blue dotted line indicates the hilum; white dotted line indicates the pericardial defect; white arrows indicate the pericardial fat pad; RA, right atrium; RV, right ventricle; LV, left ventricle; PC, pericardial cavity.

## Disclosure

The authors confirm that there is no conflict of interest.
